# Specialism and Graduate Work

**Published:** 1918-09-07

**Authors:** 


### SPECIALISM AND GRADUATE WORK.

In a rising and growing profession, such as medicine is at the present day, specialism of course multiplies; and those who, like the general physician and to a less extent the general surgeon, feel themselves threatened, raise complaints and bring charges against it, as the weaker party always will. Specialists could retaliate with tales of faulty treatment and of backwardness, but are perhaps too busy to do so. Thus study of affections of the special senses, alienism, regional surgery become more demarcated every year or two, and those who intend to engage in consultant practice in these various lines will find their way clearly marked out, if arduous and uncertain enough to follow, at various special hospitals and clinics of repute. Nothing much fresh falls to be said on this head, except that, unfortunately, for some time to come Austro-German clinics cannot be visited, and therefore greater resort will probably be had to post-graduate courses in France, Switzerland, and Copenhagen.

What, on the other hand, is of fresh and topical interest is the relation of specialism to our rapidly increasing municipal medicine; to the call there is for whole-time salaried specialists to care for the health of the working class all over the country. Tuberculosis officers, school medical officers, school oculists and dentists, infant welfare physicians, physicians for workers in dangerous trades, orthopædists—all such as these, it is pretty safe to say, will be wanted more and more as time goes on; and the more efficient they are the better it will be for everybody. Just at present one fears that they are not technically efficient. A periodical singled out recently by a leading medical contemporary is the *British Journal of Children's Diseases*: we believe very few school medical officers indeed take it. Subscriptions (no more than eight shillings a year) were asked for publicly two or three years ago, by an English tuberculosis expert, to an Italian journal on lung collapse therapy. At most three tuberculosis officers responded, and for a long time there was only one on the list. The reason for this lack of clinical keenness is mainly that clinical competence does not pay. It does not pay as regards promotion. In every way the best prospect for a school medical officer or tuberculosis officer is not to become accomplished in his specialty, pediatrics or tuberculosis, but to become a medical officer of health, whose work is purely administrative. The Public Health Medical Service is now just where the Army Medical Service was twenty years ago, before the encouragement of clinical specialism transformed and improved it: office work paid far better than ward work. As a clinical specialist, however good, the income of our S.M.I. or T.O. cannot go beyond £500 a year or at the most £600, while he remains administratively a subordinate, a junior: as a medical officer of health it may reach a thousand, and he will have a position of authority. Again, clinical competence does not even ensure election. Actual experience proves that a general practitioner in failing health may, if he has a little local influence, sell his practice and be elected to one of these posts over the head of a man with four or five years' special training. Municipal medicine then, as regards clinical appointments, is as yet a *pis aller*. And this is, of course, a very mischievous state of affairs.

Luckily, the remedies are not hard to discern, if not so easy to adopt. The present cataclysm may, however, give the opportunity. Sir William Osler has pointed out one greatly needed reform, which is to centralise the control of the anti-tuberculosis campaign. With such centralised control, rigid selection and fair election might be secured. It would be a great step forward, and would secure *standardised special post-graduate training as a necessary preliminary to appointment.*

### Private Practice for Municipal Specialists.

There remains, then, the important question of remuneration, and a very awkward one it is, in view of the tightness in money matters to be expected after the war. Now there is one excellent way of promoting clinical keenness and many other things, and that is by making income dependent thereon. If these municipal pediatricians, ophthalmologists, infant physicians, tuberculosis specialists, dental surgeons, were given their salaries but allowed in addition what some of them even now have offered them, and that is private practice, then self-interest would soon greatly raise their standard of technical competence. Such consulting practice would, of course, come to them by way of the general practitioner, with whom they are already in touch. If the practitioner saw they could be trusted with cases needing special knowledge as well as, perhaps better than, the staff of the nearest good-sized general hospital, if they could "do" refractions and squints, regulate teeth, excise scrofulous glands, induce a pneumothorax, *x*-ray bone disease, perform a mastoid operation, examine a larynx—then the long waiting-lists at provincial infirmaries would soon grow shorter, to the advantage of everybody; for even before the war the harvest field of less expensive private practice was large, but the labourers few. This, then, is the prophecy-proposal, to make municipal clinicians into hospital clinicians, doing gratis work but also private practice, and dependent for the latter upon their reputation in the former: appointed by a department—pediatrical, anti-tuberculosis, ophthalmological, orthopædic, or dental—of the now pretty certain Ministry of Health: and stunted and overshadowed no longer by local influences which it is not unfair to describe as often, in final outcome at least, distinctly corrupt. For such improved prospects many young doctors of distinction would be found willing to undergo a thorough preliminary course of post-graduate work, that foundation of all special efficiency.

